# Inhibitory effects of aprotinin on influenza A and B viruses in vitro and in vivo

**DOI:** 10.1038/s41598-021-88886-1

**Published:** 2021-05-03

**Authors:** Eun-Jung Song, Erica Españo, Sang-Mu Shim, Jeong-Hyun Nam, Jiyeon Kim, Kiho Lee, Song-Kyu Park, Chong-Kil Lee, Jeong-Ki Kim

**Affiliations:** 1 Department of Pharmacy, College of Pharmacy, Korea University, 2511 Sejong-ro, Sejong, 30019 Republic of Korea; 2 Laboratory Animal Medicine, College of Veterinary Medicine, Chonnam National University, Gwangju, 61186 Republic of Korea; 3 Division of Acute Viral Diseases, Center for Emerging Virus Research, National Institute of Health, Korea Disease Control and Prevention Agency, Cheongju, Chungbuk 28159 Republic of Korea; 4 Department of Pharmaceutics, College of Pharmacy, Chungbuk National University, Cheongju, Chungbuk 28644 Republic of Korea

**Keywords:** Drug screening, Phenotypic screening, Viral infection, Influenza virus

## Abstract

Influenza viruses cause significant morbidity and mortality worldwide. Long-term or frequent use of approved anti-influenza agents has resulted in drug-resistant strains, thereby necessitating the discovery of new drugs. In this study, we found aprotinin, a serine protease inhibitor, as an anti-influenza candidate through screening of compound libraries. Aprotinin has been previously reported to show inhibitory effects on a few influenza A virus (IAV) subtypes (e.g., seasonal H1N1 and H3N2). However, because there were no reports of its inhibitory effects on the other types of influenza viruses, we investigated the inhibitory effects of aprotinin in vitro on a wide range of influenza viruses, including avian and oseltamivir-resistant influenza virus strains. Our cell-based assay showed that aprotinin had inhibitory effects on seasonal human IAVs (H1N1 and H3N2 subtypes), avian IAVs (H5N2, H6N5, and H9N2 subtypes), an oseltamivir-resistant IAV, and a currently circulating influenza B virus. We have also confirmed its activity in mice infected with a lethal dose of influenza virus, showing a significant increase in survival rate. Our findings suggest that aprotinin has the capacity to inhibit a wide range of influenza virus subtypes and should be considered for development as a therapeutic agent against influenza.

## Introduction

Influenza viruses remain important pathogens that cause respiratory diseases in humans and animals. Human seasonal influenza A and B viruses annually cause severe morbidity worldwide. The Centers for Disease Control (CDC) estimates around 23,000 flu-related deaths in the United States each year^1^. In addition, avian influenza viruses, such as the H5 and H7 subtypes, sporadically cause highly lethal infections in both animals and humans^2–4^, and animal or human-animal influenza reassortant strains occasionally cause global epidemics or pandemic influenza^5^.

Vaccination is considered the most effective strategy for controlling influenza in humans^6^. However, current influenza vaccines have several limitations, including their limited efficacy due to antigenic mismatches between the vaccine and circulating virus strains^7^. For this reason, antiviral drugs are important for controlling influenza. Representative classes of anti-influenza drugs include adamantane-based matrix protein 2 (M2) ion channel blockers (e.g., amantadine and rimantadine) and neuraminidase (NA) inhibitors (e.g., oseltamivir and zanamivir)^8^. However, the emergence of antiviral drug resistance is a constant concern, owing to the high mutation rates of influenza viruses through antigenic drift and shift^9^. Since the first report of amantadine-resistant influenza A viruses (IAVs) during the 1980 epidemic^10^, the prevalence of these viruses among circulating IAVs (especially, H1N1 and H3N2 subtypes) has increased rapidly to nearly 100% of the cases^11^. In response, the CDC has stopped recommending the use of adamantane in the United States^12^. Increasing application of NA inhibitors (especially oseltamivir) brings into focus the risk of developing resistance to this class of anti-influenza drugs. Although the prevalence of NA inhibitor-resistant influenza viruses is generally low (oseltamivir < 3.5%) or rare (zanamivir < 1%)^13–16^, the problem of reduced susceptibility and resistance of influenza viruses to NA inhibitors has been recently increasing. Therefore, it is an utmost need to develop better or novel anti-influenza drugs to prepare for emergencies.

In this study, we first aimed to identify anti-influenza viral agents by screening compound libraries. Aprotinin, a serine protease inhibitor used to prevent bleeding in cardiopulmonary bypass surgery, presented as a candidate. Previous reports have suggested that aprotinin has anti-influenza virus activity based on its ability to prevent the cleavage of hemagglutinin (HA), a step required for viral-host fusion^17–19^. However, most reports cover only a narrow range of IAV strains (especially seasonal IAV strains) and strains of influenza B virus (IBV) that may no longer be circulating. Furthermore, little is known about the effects of aprotinin against oseltamivir-resistant IAV strains.

Therefore, in this study, we investigated the effects of aprotinin on various subtypes of IAV, including (i) human seasonal IAVs, (ii) avian influenza viruses with zoonotic potential (H5N2, H9N2, and H6N2), (iii) oseltamivir-resistant IAV, and (iv) on a currently circulating strain of IBV in vitro. We also used a mouse model to verify the anti-influenza activity of aprotinin. Our findings contribute further evidence to the potential of aprotinin as a broad-spectrum anti-influenza agent.

## Results

### In vitro dose-dependent inhibitory effects of aprotinin on influenza A and B viruses

**Figure 1:**
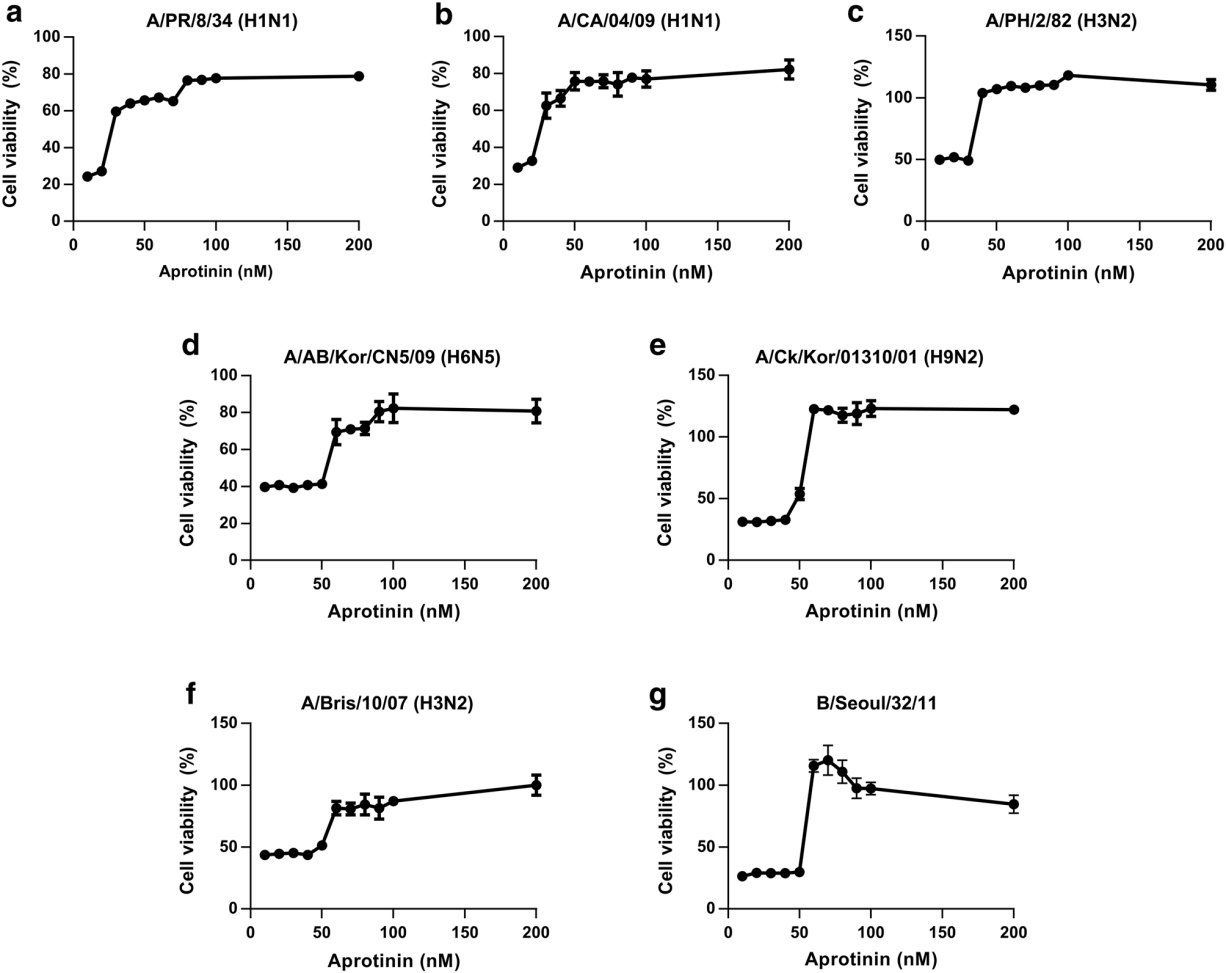
Dose-dependent effects of aprotinin against influenza viruses in vitro. Madin–Darby canine kidney (MDCK) cells were infected with (**a**) A/PR/8/34 (H1N1), (**b**) A/CA/04/09 (H1N1), (**c**) A/PH/2/82 (H3N2), (**d**) A/AB/Kor/CN5/09 (H6N5), (**e**) A/Ck/Kor/01310/01 (H9N2), (**f**) A/Bris/10/07 (H3N2), or (**g**) B/Seoul/32/11 (Yamagata-like lineage) and were treated with varying concentrations of aprotinin (10‒200 nM; n = 3 per dose) for 72 h. Viruses were inoculated at a dose of 100 (H6N5 and B/Seoul) or 1000 (all except H6N5 and B/Seoul) TCID_50_ per well. Cell viability was measured using the EZ-Cytox reagent, and cell viability was calculated relative to the uninfected MDCK cell viability (cell-only control). TCID_50_: median tissue culture infectious dose.

To develop better or novel antiviral drugs against influenza virus infection, we screened compound libraries through a Madin–Darby canine kidney (MDCK) cell-based screening system^20^ using A/PR/8/34 (H1N1) virus, the standard reference strain of human IAV. We tested a total of 1280 compounds and found 13 anti-influenza candidates. Aprotinin, a serine protease inhibitor, was selected as a final candidate because both aprotinin samples from this library and from that of another company showed inhibitory effects on the virus (data not shown). To validate the results of screening, we treated MDCK cells infected with different influenza virus strains with varying concentrations (10‒200 nM) of aprotinin (Fig. 1). Viral inhibition assay in MDCK cells showed that aprotinin inhibits A/PR/8/34 (H1N1) in a dose-dependent manner (Fig. 1a). Aprotinin could also inhibit A/CA/04/09 (H1N1), A/PH/2/82 (H3N2), A/AB/Kor/CN05/09 (H6N5), A/Ck/Kor/01310/01 (H9N2), A/Bris/10/07 (H3N2), and B/Seoul/32/11 in a dose-dependent manner (Fig. 1b–g). The calculated EC_50_ values for aprotinin against the different influenza strains are shown in Table 1. Based on the results of the viral inhibition assay, we applied the lowest effective concentration against all the tested influenza strains (60 nM) for subsequent experiments.

**Table 1:** Half-maximal effective concentrations (EC_50_) of aprotinin against various influenza virus strains in Madin–Darby canine kidney (MDCK) cells.

Type	Virus	EC_50_
A	A/PR/8/34 (H1N1)	14 nM
A	A/CA/04/09 (H1N1, 2009 pandemic)	11 nM
A	A/PH/2/82 (H3N2)	21 nM
A	A/AB/Kor/CN5/09 (H6N5)	87 nM
A	A/Ck/Kor/01310/01 (H9N2)	57 nM
A	A/Bris/10/07 (H3N2, oseltamivir-resistant)	110 nM
B	B/Seoul/32/11 (Yamagata-like)	39 nM

**Figure 2:**
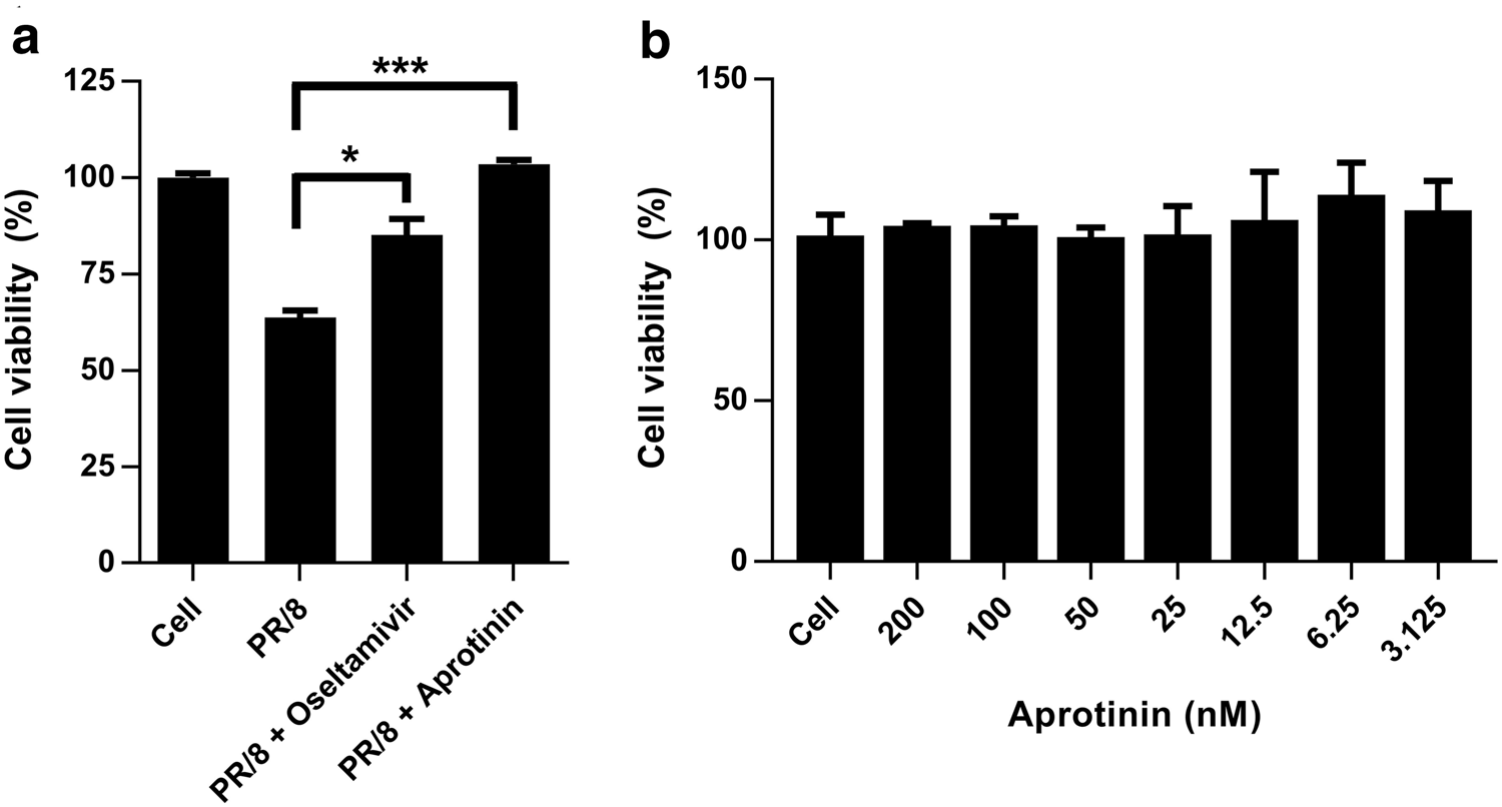
Antiviral effects of aprotinin compared with oseltamivir and cytotoxicity assay in Madin–Darby canine kidney (MDCK) cells. (**a**) MDCK cells were infected with A/PR/8/34 (1000 TCID_50_/ml) and treated with 60 nM aprotinin or 100 μM oseltamivir. Untreated and uninfected MDCK cells (cell) and untreated infected cells (PR/8) were used as controls. (**b**) To determine the cytotoxicity of aprotinin, cell viability was measured by treating the MDCK cells with the compound for 72 h, and cell viability was compared with untreated control cells (cell). Cell viability was measured using EZ-Cytox. The experiments were performed in triplicate. *P < 0.05, **P < 0.01, and ***P < 0.001. TCID_50_: median tissue culture infectious dose.

We next compared the antiviral activity of aprotinin against A/PR/8/34 (H1N1) virus with that of oseltamivir (100 μM). Aprotinin showed corresponding or superior antiviral activity to oseltamivir against PR/8 virus infection (Fig. 2a). Colorimetric cytotoxicity assay revealed that there was no cytotoxicity in the range of aprotinin concentrations tested in this study (≤ 200 nM) (Fig. 2b).

### In vitro inhibitory effects of aprotinin on multiple replication cycles of various influenza A virus subtypes

Previous studies have minimal information on the spectrum of the anti-influenza viral activity of aprotinin. Therefore, we evaluated the effects of aprotinin treatment on the production of infectious particles of various IAV subtypes, including human and avian viruses, through time-based studies by determining the growth kinetics of the following viruses in MDCK cells: A/PR/8/34 (H1N1), A/CA/04/09 (H1N1), A/PH/2/82 (H3N2), A/AB/Kor/CN2/09 (H5N2), A/AB/Kor/CN5/09/H6N5 (H6N5), and A/Ck/Kor/01310/09 (H9N2) viruses. Culture supernatants were collected at different time points, and virus titers were determined by calculating the median TCID_50_ based on the hemagglutination assay. We also compared the inhibitory effects of aprotinin against those of oseltamivir (100 μM).

**Figure 3:**
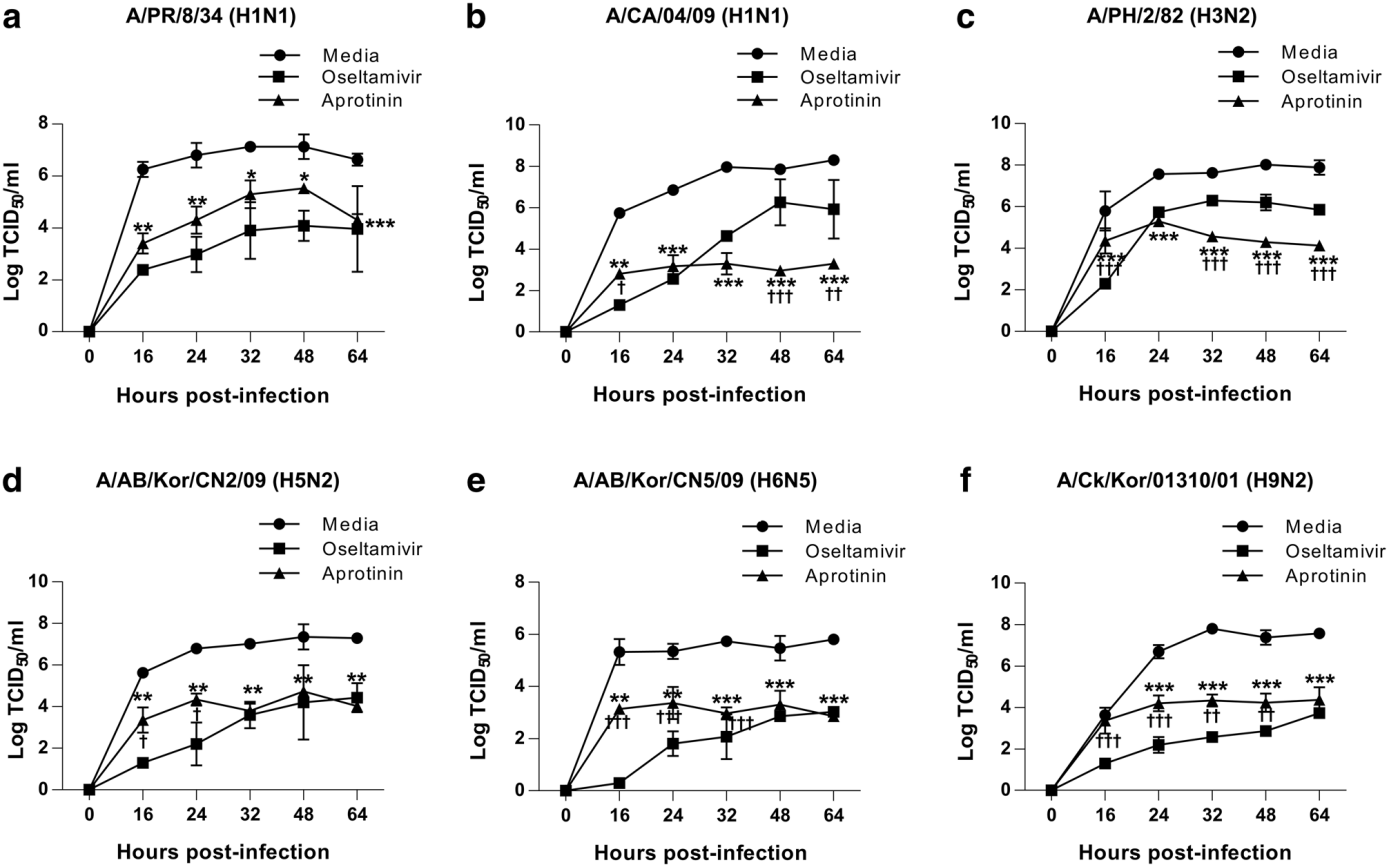
Aprotinin inhibited the replication of various strains of human influenza A virus in Madin–Darby canine kidney (MDCK cells. The replication kinetics of (**a**) A/PR/8/34 (H1N1), (**b**) A/CA/04/09 (H1N1), (**c**) A/PH/2/82 (H3N2), (**d**) A/AB/Kor/CN2/09 (H5N2), (**e**) A/AB/Kor/CN5/09 (H6N5), and (**f**) A/Ck/Kor/01310/01 (H9N2) virus were investigated in MDCK cells after treatment with aprotinin and oseltamivir. MDCK cells were infected with influenza virus at an MOI of 0.001 (H6N5) or 0.01 (all viruses except H6N5) for 1 h and then treated with aprotinin (60 nM) or oseltamivir (100 μM). Supernatants were collected pre-infection (0) and at 16, 24, 32, 48, and 64 h post-infection, and viral titers in the supernatants were determined by the TCID_50_ assay. *P < 0.05, **P < 0.01, and ***P < 0.0001, statistically significant difference between the virus-only (media) group and the aprotinin treatment group. ^**†**^*P* < 0.05, ^**††**^*P* < 0.01, and ^**†††**^*P* < 0.0001, statistically significant difference between the aprotinin and oseltamivir treatment group. MOI: multiplicity of infection; TCID_50_: half-maximal tissue culture infectious dose.

Aprotinin was able to significantly reduce the production of the tested human IAVs after more than 16 h post-infection (Fig. 3a–c), supporting the results of previous studies^18, 19^. Furthermore, the inhibitory effects of aprotinin on A/CA/04/09 (H1N1) and A/PH/2/82 (H3N2) viruses were more superior than those of oseltamivir towards the end of the incubation period. Aprotinin was also able to inhibit the production of avian influenza viruses in MDCK cells (Fig. 3d–f). Aprotinin displayed weaker inhibitory effects on avian IAVs than oseltamivir at early time points post-infection but presented effects similar to those of oseltamivir at 64 h post-infection. These results indicate that aprotinin had inhibitory effects on infections of both human and avian IAVs.

### In vitro inhibitory effects of aprotinin on multiple cycles of replication of oseltamivir-resistant influenza A and B viruses

**Figure 4:**
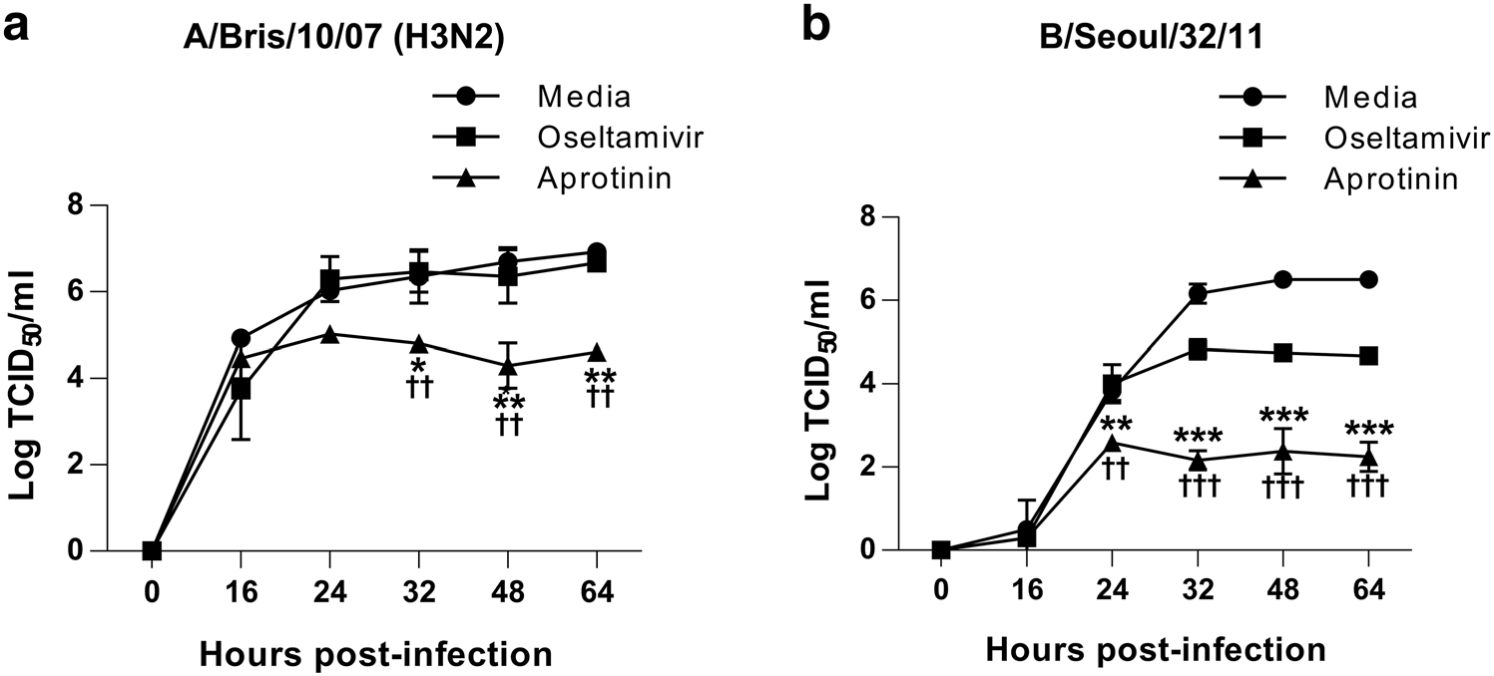
Aprotinin inhibited the replication of oseltamivir-resistant influenza viruses in Madin–Darby canine kidney (MDCK) cells. The replication kinetics of (**a**) A/Bris/10/07 (H3N2) and (**b**) B/Seoul/32/11 were investigated in MDCK cells after treatment with aprotinin and oseltamivir. MDCK cells were infected with influenza virus at an MOI of 0.01 (H3N2) or 0.001 (B/Seoul/32/11) for 1 h and then treated with aprotinin (60 nM) or oseltamivir (100 μM). Supernatants were collected pre-infection (0) and at 16, 24, 32, 48, and 64 h post-infection, and viral titers in the supernatants were determined by the TCID_50_ assay. *P < 0.05, **P < 0.01, and ***P < 0.0001, statistically significant difference between the virus-only (media) group and the aprotinin treatment group. ^**†**^*P* < 0.05, ^**††**^*P* < 0.01, and ^**†††**^*P* < 0.0001, statistically significant difference between the aprotinin and oseltamivir treatment groups. MOI: multiplicity of infection; TCID_50_: half-maximal tissue culture infectious dose.

We next examined the inhibitory effects of aprotinin against an oseltamivir-resistant IAV (A/Bris/10/07; H3N2). As shown in Fig. 4a, A/Bris/10/07 (H3N2) virus was less susceptible to oseltamivir. Our genetic analysis revealed that the H28T mutation in HA conferred the reduced susceptibility of the H3N2 virus to oseltamivir (data not shown). However, aprotinin could effectively reduce the production of the H3N2 virus (Fig. 4a).

IBVs are generally less susceptible to oseltamivir than IAVs^21, 22^. Therefore, we investigated the inhibitory effects of aprotinin on influenza B virus (B/Seoul/32/2011) infection. As shown in Fig. 4b, the IBV was around 50- to 100-fold less susceptible to oseltamivir. However, aprotinin was more effective than oseltamivir at reducing IBV production.

Taken together, these results suggest that aprotinin can significantly reduce the production of oseltamivir-resistant IAV and IBV.

### Inhibitory effects of aprotinin treatment in mice infected with a lethal dose of A/PR/8/34 (H1N1) virus

To test whether aprotinin has antiviral activity in vivo, we tested its effects against lethal A/PR/8/34 (H1N1) virus infection in C57BL/6 mice. We initially treated mice with once-daily intravenous injections of aprotinin at 2 mg/kg mouse body weight per day based on a previous study^23^. However, while it did not have toxic effects in vivo, it also did not display antiviral effects in influenza-infected mice (data not shown). The initial half-life of aprotinin may be too short for conferring antiviral effects in vivo. As such, we decided to administer aprotinin twice a day.

**Figure 5:**
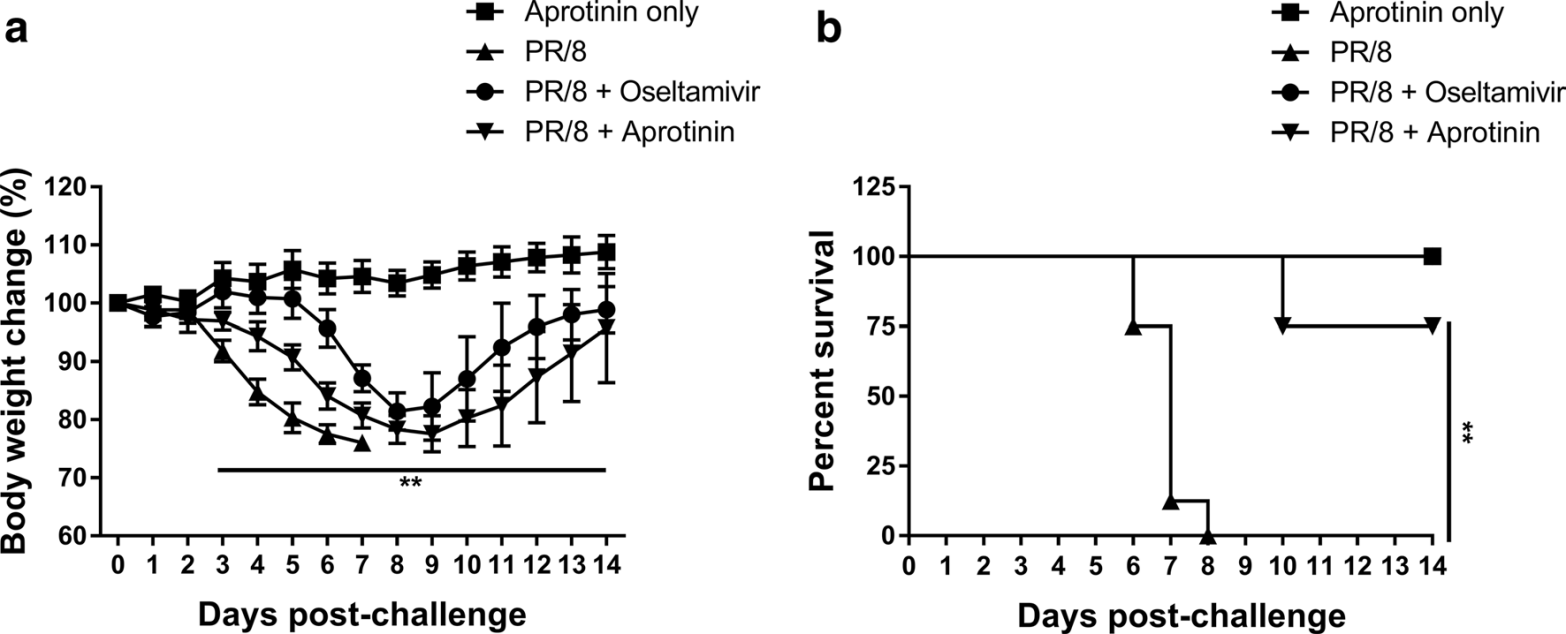
Antiviral effects of aprotinin against influenza A virus in C57BL/6 mice. Groups of mice (n = 8 per group) were intranasally infected with A/PR/8/34 (H1N1) virus at three times the 50% mouse lethal dose (3 LD_50_). Oseltamivir was orally administered (oral gavage) at 10 mg/kg/day, and aprotinin was intravenously administered at 2 mg/kg, twice daily, for 5 days. Mouse (**a**) body weight changes and (**b**) survival were monitored daily for 2 weeks. ** *P* < 0.01, significant difference between the negative control group (virus-infected only, PR/8) and the aprotinin treatment group (PR/8 + aprotinin).

C57BL/6 mice were intranasally inoculated with 3 LD_50_ of A/PR/8/34 (H1N1) virus. For 5 days after infection, the mice received twice-daily intravenous injections of aprotinin (2 mg/kg mouse body weight, twice daily); oral administrations of oseltamivir (10 mg/kg/day) based on a previously reported effective dose^24^; or phosphate-buffered saline (PBS) as control. Uninfected mice were similarly treated with aprotinin as the drug-only control group. Body weight changes and survival were monitored daily for 14 days after infection (Fig. 5). Administration of aprotinin alone did not result in body weight loss in mice. The PBS-treated mice had 0% survival at 8 days post-infection. Meanwhile, the groups of mice treated with either aprotinin and oseltamivir showed 75% and 100% survival, respectively.

## Discussion

Given the limitations of influenza vaccines and the recent rise in the number of oseltamivir-resistant strains, there remains a need to discover and develop new anti-influenza agents. In our cell culture-based screening of compound libraries, aprotinin was identified as a strong anti-influenza candidate. Aprotinin, also known as bovine pancreatic trypsin inhibitor, is a naturally occurring non-specific inhibitor of serine proteases, including trypsin, chymotrypsin, plasmin, and kallikrein^25^. It is primarily indicated for preventing blood loss in cardiac bypass surgeries. It was considered to be well-tolerated in animal models and in clinical trials^26^. Aprotinin has been previously reported as an anti-influenza agent in vitro^27^, in embryonated chicken eggs^17, 28^, and in mice^29^. It is currently licensed in Russia for clinical use in aerosolized form (Aerus), primarily against seasonal H1N1 and H3N2 influenza, but it has also been tested against H2N2, pandemic H1N1, and avian-like H7N9 influenza viruses^18, 19, 28^.

Influenza viruses require proteolytic cleavage and structural rearrangement of HA for successful fusion with host endosomes. HA is initially translated as a precursor, HA0, with HA1 and HA2 domains linked by a short peptide sequence. Trypsin-like proteases facilitate cleavage of the precursor by targeting arginine in the linker peptide in the HA0 of most influenza virus strains. Extracellular cleavage of the HA0 of mammalian and low-pathogenicity influenza viruses is facilitated by soluble proteases such as tryptase Clara, mini-plasmin, and ectopic anionic trypsin I^30^. Cell-associated cleavage of HA0 in the human airway can also be facilitated by transmembrane protease serine 2 (TMPRSS2) in secretory vesicles during viral egress and by the membrane-bound human airway-trypsin like protease (HAT) prior to attachment to target cells^30, 31^. Aprotinin inhibits the activity of trypsin-like proteases by blocking the active site, thereby inhibiting proteolytic cleavage of HA0^28^. Aprotinin also displayed inhibitory effects on paramyxoviruses^32^ and SARS-CoV-2^33^, likely due to suppressed cleavage of the fusion protein precursor and the spike protein, respectively.

Because previous studies have shown that aprotinin inhibited a limited number of subtypes of IAV (mainly H1N1 and H3N2), we decided to examine its antiviral activity against a broader range of influenza viruses in this study. The tested strains include avian strains of IAV, an oseltamivir-resistant strain of IAV, and a currently circulating strain of IBV.

Similar to previous reports, we found that aprotinin was able to inhibit the production of seasonal H1N1 and H3N2 IAVs in MDCK cells. The effects of aprotinin were either comparable or superior to the effects of oseltamivir. We also found that aprotinin could inhibit avian IAVs belonging to the H9N2, H5N2, and H6N5 subtypes in vitro at levels similar to the effects of oseltamivir. H9N2 currently circulates in poultry and is generally avirulent or low-pathogenic. However, occasional outbreaks in poultry farms have occurred, and sporadic human infection cases have also been reported^34^. Meanwhile, both the H5N2 and H6N5 viruses in this study were isolated from wildfowl in South Korea. The H6N5 isolate was found to cause considerable morbidity and mortality in mice without bearing any known pathogenicity marker^35^, and the H5N2 isolate adapted to and caused lethality in mice after only a single lung-to-lung passage^36^. These previous studies suggest that some of the currently circulating avian influenza virus strains have the capacity to easily cross the avian-mammalian transmission barrier and may emerge as zoonotic agents in the future. The ability of aprotinin to inhibit these avian influenza viruses suggests that aprotinin may potentially be used in human outbreaks of avian influenza viruses.

We have also shown that aprotinin is able to inhibit an oseltamivir-resistant influenza A strain (A/Bris/10/07; H3N2). Additionally, similar to earlier reports of aprotinin’s activity against the B/Lee/40 and B/HK/73 viruses^17^, aprotinin shows antiviral activity against a currently circulating strain of IBV (Yamagata-like lineage, B/Seoul/32/2011). IBVs are generally less susceptible to oseltamivir, especially in children^21, 37^. Because aprotinin targets a host factor required for infection, influenza viruses are less likely to develop aprotinin resistance, especially because trypsin-like proteases, the targets of aprotinin, are required for influenza virus proliferation. Therefore, the use of aprotinin may be more beneficial in the long run than the use of drugs targeted against viral components.

In our study, at least twice-daily intravenous administrations were needed for aprotinin to be protective against influenza virus infection in a mouse model. Aprotinin has a relatively short plasma half-life (0.7–2 h), and 90% of the administered dose is absorbed by the kidney in a few hours^38^, which requires high-dose intravenous administrations of aprotinin in surgeries^26^. This probably explains why once-daily intravenous administrations were not sufficient to exert inhibitory effects against the influenza virus. As such, high plasma concentrations of aprotinin may also be required to inhibit influenza viruses. However, as in the case of the licensed aerosolized aprotinin in Russia, multiple doses of intranasally administered aprotinin may be more beneficial for application against influenza virus infection in humans^18, 19, 29, 39^. This way, aprotinin does not have to circulate systemically and will be targeted in the upper respiratory tract, where most influenza virus subtypes replicate in humans. However, in this study, we did not test intranasal administration of aprotinin. Future studies will have to be performed to determine the optimal dosage and route of administration for human application. Moreover, we did not measure viral titers from the mouse respiratory tract and lungs. Testing the effects of aprotinin on titers of different influenza virus subtypes in the mouse respiratory tract should be considered for further studies. Additionally, whether aprotinin will be effective against highly pathogenic avian influenza viruses (HPAIVs) will have to be evaluated. HPAIVs have multibasic cleavage sites that are more accessible to a wide range of proteases^40^. If aprotinin has the ability to inhibit HPAIVs, then it will be a viable pandemic influenza therapeutic candidate that runs a lower risk of causing drug resistance than currently used antivirals like oseltamivir.

Several studies have associated the use of aprotinin in cardiac bypass surgery with risks of renal failure and mortality^41–43^. Furthermore, re-exposure to aprotinin within six months has been associated with allergic reactions, including anaphylaxis, with a peak at 4–30 days from last exposure^44^. Consequently, aprotinin was pulled out of the market in 2007. In surgery, aprotinin is typically intravenously administered at an initial dose of 1.0–2.0 × 10^6^ kallikrein inhibitor units (KIU), followed by continuous infusion with 0.25–0.5 × 10^6^ KIU/hour^45^. Zhirnov et al. reported that 1.0–3.0 × 10^3^ KIU per day of aerosolized aprotinin was sufficient against influenza virus infection^19^. Given the nearly 1000-fold difference in dose and the different routes of administration between the application of aprotinin in cardiac bypass surgery and in influenza treatment, subsequent adverse effects may also differ. Notably, Zhirnov et al. reported that aerosolized aprotinin did not cause allergic reactions in an early clinical trial^19^. Furthermore, several groups have challenged the existing evidence on the adverse effects of aprotinin and have shown that aprotinin does not independently increase the risk of renal failure and mortality^45–48^. Indeed, after reviewing evidence on the benefits and risks of aprotinin, Canada and Europe have re-licensed aprotinin for application in cardiac bypass surgery^45, 48^. Thus, with the potentially broad applicability of aprotinin against influenza viruses and the potential differences in dosage requirements, we believe that further evaluation of aprotinin as a treatment for influenza is merited despite the reported adverse effects for the original indication of aprotinin.

Taken together, in this study, we were able to demonstrate that aprotinin inhibits the in vitro production of (1) avian IAVs with zoonotic potential, (2) oseltamivir-resistant IAV, and (3) currently circulating IBV, which is inherently less susceptible to oseltamivir. Thus, we propose that aprotinin is an excellent candidate for the treatment of most IAVs and IBVs in humans. However, whether aprotinin is similarly effective against HPAIVs will have to be evaluated. The most appropriate route of administration and the optimal dosage for the clinical use of aprotinin against influenza will also have to be determined.

## Materials and methods

### Cells, viruses, and aprotinin

Madin–Darby canine kidney (MDCK) cells were cultured in Growth Medium: 1 × Minimum Essential Medium (Invitrogen, Carlsbad, CA, USA) supplemented with 10% fetal bovine serum (FBS; Gibco, Thermo Fisher Scientific Inc., Waltham, MA, USA), 3% L-glutamine (Gibco), 0.75% sodium bicarbonate (Gibco), 1% MEM vitamin solution (Sigma-Aldrich, St. Louis, MO, USA), 50 μg/ml gentamicin (Gibco), and 1% antibiotic–antimycotic solution (Gibco).

Seven IAVs and one IBV were used in this study: A/Puerto Rico/8/1934 (A/PR/8/34; H1N1); A/California/04/2009 (A/CA/04/09; H1N1); A/Philippines/2/1982 (A/PH/2/82; H3N2); A/Brisbane/10/2007 (A/Bris/10/07; H3N2); A/Aquatic Bird/Korea/CN2/2009 (A/AB/Kor/CN2/09; H5N2); A/Aquatic Bird/Korea/CN5/2009 (A/AB/Kor/CN5/09; H6N5); A/Chicken/Korea/01310/2001 (A/Ck/Kor/01310/01; H9N2); and B/Seoul/32/2011 (B/Seoul/32/11). The IAVs were grown in 10-day-old embryonated chicken eggs for 48 h at 35 °C. The allantoic fluid was harvested, and aliquots were stored at ‒70 °C until use. The IBV was propagated in MDCK cells in Infection Medium: 1 × MEM supplemented with 0.3% bovine serum albumin (Sigma-Aldrich) (instead of FBS) and 1.0 μg/ml tosylsulfonyl phenylalanyl chloromethyl ketone (TPCK)-trypsin (Worthington Biochemical Corporation, Lakewood, NJ, USA) incubated at 37 °C, 5% CO_2_. After incubation for 72 h, the supernatant was harvested, and aliquots were stored at ‒ 70 °C until use.

Aprotinin (Sigma-Aldrich) was dissolved in distilled water to make stock solutions, and aliquots were stored at ‒ 20 °C until use. Oseltamivir phosphate (Tamiflu; Roche, Basel, Switzerland) was reconstituted in PBS and used on the same day. Working solutions of both aprotinin and oseltamivir were prepared by diluting the stock solutions with Infection Medium on the day of use for in vitro assays and with PBS for the in vivo study.

### Cell viability and cytotoxicity assays

For the cell viability assay, MDCK cells were cultured on a 96-well plate (2 × 10^4^ cells/well) in Growth Medium. After incubation for 16 h at 37 °C, the cells were infected with each influenza virus at 100 or 1000 half-maximal tissue culture infectious dose (TCID_50_)/well and were washed with phosphate-buffered saline at 1 h post-infection. Various concentrations (10 to 200 nM, n = 3 per dose) of aprotinin diluted with Infection Medium (similar to the IBV infection medium, with TPCK-trypsin) were added into each well to a final volume of 100 μl/well. After incubation for 72 h at 37 °C, cell viability was determined using the EZ-Cytox kit (Daeillab Service Co., South Korea) according to the manufacturer’s instructions. Cell viability was indicated by percentage values compared to the negative control (cells that were infected but not treated with aprotinin). Cytotoxicity of aprotinin was measured similarly as described above, but without infection. Cytotoxicity was presented as % cell viability relative to the negative control (wells containing cells only).

### Virus growth kinetics

MDCK cells were cultured on a 24-well plate (1.25 × 10^5^ cells/well) in Growth Medium. After incubation for 16 h at 37 °C, the cells were infected with each influenza virus at a multiplicity of infection (MOI) of 0.01 or 0.001 and were washed with PBS at 1 h post-infection. The cells were treated with aprotinin (60 nM/well) or oseltamivir (100 μM/well; positive control) in a total volume of 0.5 ml Infection Medium/well and incubated at 37 °C, 5% CO_2_. Culture supernatant was collected pre-infection and at 16, 24, 32, 48, and 64 h post-infection and stored at ‒ 70 °C until analysis.

Virus titration was performed using MDCK cells. The cells were cultured on a 96-well plate (2 × 10^4^ cells/well) in Growth Medium and were infected with 100 μl of serial tenfold dilutions of the culture supernatant in Infection Medium. After incubation for 72 h at 37 °C, the culture supernatant was harvested to determine virus titration by the hemagglutination assay using 0.5% chicken red blood cells. The virus titers were determined by calculating the TCID_50_ using the Reed-Muench method^49^. The typical limit of detection for the TCID_50_ assay of influenza viruses on MDCK cells is 10^0.3^ TCID_50_/ml.

### Mouse experiments

All animal experiments were conducted in biosafety level 2-plus facilities at the Korea Research Institute of Bioscience and Biotechnology (KRIBB; Daejeon, South Korea). The study was approved by the Institutional Animal Care and Use Committee (IACUC) of KRIBB (approval no. KRIBB-AEC-10046). Animal care and experiments were carried out according to the guidelines of KRIBB IACUC. The studies were designed and are reported in line with the Animal Research: Reporting of In Vivo Experiments (ARRIVE) guidelines.

Six- to eight-week-old C57BL/6 mice were purchased from Koatech (Pyeongtaek, South Korea), and eight mice per group were used. Each mouse was anesthetized with 100 μl of 1:1:8 Zoletil:Rompun:PBS (intraperitoneal). Then, the mice were inoculated via intranasal instillation with 3 times the 50% lethal dose (3 LD_50_) of the PR/8 virus in PBS (30 μl/mouse). The following day, the mice were treated intravenously with aprotinin in PBS (2 mg/kg mouse body weight, twice daily; 0.1 ml/mouse); orally (oral gavage) with oseltamivir in PBS (10 mg/kg mouse body weight per day; 0.2 ml/mouse), or intravenously with PBS (negative control) twice a day for 5 days. Uninfected mice were likewise intravenously administered aprotinin in PBS as the aprotinin-only control group. The mice were monitored daily for 14 days for weight change and mortality.

### Data analysis

Based on the inhibition of virus growth, the half-maximal effective concentration (EC_50_) of aprotinin was calculated using GraphPad Prism (version 5; San Diego, CA, USA). This procedure is commonly known as a logistic regression using the following formula: Y = 1/1 + 10 ([logEC_50_ – logX] × Hillslope), where Y represents response (inhibition of virus growth), and X represents the concentration of aprotinin. Hillslope is the parameter to describe the steepness of the curve. Data analysis was performed using GraphPad Prism for Windows. Student’s *t*-test was performed to determine differences between two groups. For the virus growth kinetics data, significant differences among the groups were evaluated by ANOVA followed by Tukey’s multiple comparison test. The Gehan–Breslow–Wilcoxon test was used to analyze differences in mouse survival. *P* values less than 0.05 (*P* < 0.05) were considered statistically significant.
